# *Cis*- and *Trans*-Acting Elements Regulate the Mouse *Psmb9* Meiotic Recombination Hotspot 

**DOI:** 10.1371/journal.pgen.0030100

**Published:** 2007-06-22

**Authors:** Frédéric Baudat, Bernard de Massy

**Affiliations:** Institute of Human Genetics, Centre National de la Recherche Scientifique, Unité Propre de Recherche 1142, Montpellier, France; Stowers Institute for Medical Research, United States of America`

## Abstract

In most eukaryotes, the prophase of the first meiotic division is characterized by a high level of homologous recombination between homologous chromosomes. Recombination events are not distributed evenly within the genome, but vary both locally and at large scale. Locally, most recombination events are clustered in short intervals (a few kilobases) called hotspots, separated by large intervening regions with no or very little recombination. Despite the importance of regulating both the frequency and the distribution of recombination events, the genetic factors controlling the activity of the recombination hotspots in mammals are still poorly understood. We previously characterized a recombination hotspot located close to the *Psmb9* gene in the mouse major histocompatibility complex by sperm typing, demonstrating that it is a site of recombination initiation. With the goal of uncovering some of the genetic factors controlling the activity of this initiation site, we analyzed this hotspot in both male and female germ lines and compared the level of recombination in different hybrid mice. We show that a haplotype-specific element acts at distance and in *trans* to activate about 2,000-fold the recombination activity at *Psmb9*. Another haplotype-specific element acts in *cis* to repress initiation of recombination, and we propose this control to be due to polymorphisms located within the initiation zone. In addition, we describe subtle variations in the frequency and distribution of recombination events related to strain and sex differences. These findings show that most regulations observed act at the level of initiation and provide the first analysis of the control of the activity of a meiotic recombination hotspot in the mouse genome that reveals the interactions of elements located both in and outside the hotspot.

## Author Summary

In most sexually reproducing species, during meiosis a high level of recombination between homologous chromosomes is induced. These events are not evenly distributed in the genome but clustered in small regions called hotspots. The genetic factors controlling their activity in mammals are still poorly understood. We have performed experiments to identify factors that influence the recombination activity of a hotspot in the mouse genome. By detecting the recombination products by a PCR-based method, we show that the variation of hotspot activity (up to 2,000-fold) is mainly due to differences of initiation frequencies, rather than differences at later steps of recombination. In addition, we identify several levels of controls. First, the initiation of recombination is activated by a haplotype-specific element, localized outside the hotspot and acting in *trans* (when heterozygous, this element allows for recombination initiation on both homologous chromosomes). This suggests a unique type of regulation requiring the presence of a diffusible factor and/or of communications between homologous chromosomes before recombination. A second element represses the recombination initiation in *cis,* which might indicate the influence of local polymorphisms affecting initiation events. Our results provide the first functional analysis of the control of recombination initiation sites for meiotic recombination in mammals.

## Introduction

In most eukaryotes, the formation of at least one reciprocal exchange, or crossover (CO), per chromosome pair between nonsister chromatids provides the physical connection that is required for the segregation of homologous chromosomes at the first meiotic division. The molecular mechanism of meiotic recombination has been described in Saccharomyces cerevisiae. Recombination is initiated by the formation of DNA double-strand breaks (DSBs), catalyzed by Spo11 [1]. These DSBs are repaired by interactions with a nonsister chromatid, giving rise to two types of homologous recombination products, CO and gene conversion without associated crossover (NCO). Both types of recombination events have in common the gene conversion of the sequences surrounding the DSB site. Despite the fact that both events are initiated by Spo11-dependent DSBs, formations of CO and NCO have different genetic requirements and are thought to involve different molecular intermediates [2,3]. Therefore, both the distribution of DSBs and the local variation in the proportion of events giving rise to a CO contribute to the final distribution of CO along the genome. Among the strongest evidence in favor of the conservation of the molecular mechanism of meiotic recombination among eukaryotes is the wide conservation of Spo11, which has been found to be required for meiotic recombination in all organisms in which it has been tested [1]. A number of other proteins involved in later steps in the process of meiotic recombination are also structurally and functionally conserved [4,5].

Meiotic recombination events are not distributed evenly within the genome, but vary both locally and on a large scale. An important feature of local variation is the presence of recombination hotspots, which are defined as intervals where the recombination rate is significantly higher than in neighboring intervals. In S. cerevisiae recombination hotspots result from the clustering of initiating DSBs at localized preferential sites, spreading over 50–500 bp. The recombination events, both CO and NCO, extend in short intervals (<4 kb) surrounding these initiation sites (reviewed in [6]). Most recombination events take place at these hotspots, as shown by the correlation of the genetic map with the distribution of meiotic DSBs [7–10]. Sperm-typing analyses of a few mouse and human CO hotspots revealed a structure reminiscent of the distribution of recombination events in yeast hotspots: CO are clustered over 2 kb or less, with a density that peaks at hotspot centers and decreases on both sides (reviewed in [11,12]). A high frequency of gene conversion without reciprocal exchange (NCO) has been detected at the mouse *Psmb9* hotspot and at several human hotspots, demonstrating that they correspond to sites of recombination initiation [13–16]. Consistent with the hypothesis of CO hotspots as initiation sites, DNA breaks have been detected in testes at the mouse *Ea* CO hotspot [17]. Several observations show that most recombination is concentrated in hotspots in mammals as well as in yeast. In the mouse major histocompatibility complex (MHC), five to eight CO hotspots have been identified by pedigree analyses, accounting for most of the CO detected in this region [18,19]. In human, the distribution of CO has been analyzed in a 292-kb interval in the class II region of MHC by population studies, which were completed by sperm-typing analyses over short selected intervals. These studies revealed that the vast majority of the CO occurring in this region localize at seven hotspots [20,21]. Similarly, the majority of CO is clustered in short hotspot intervals also in intervals located outside the MHC, as shown by one pedigree analysis in mouse and a few sperm-typing analyses in men [22–24]. These conclusions have been extended recently to the whole human genome by genome-wide determination of historical CO rates on the basis of population analysis [25,26]. With this methodology, Myers et al. [26] came to the conclusion that most CO occur in hotspots: They estimated that 80% of all recombination occurs in 10%–20% of the human genome and identified more than 25,000 CO hotspots.

The factors controlling the activity of recombination hotspots remain elusive. One approach has been to search for features related to the DNA sequence by comparing the genome-wide distribution of hotspots with the variation of various features along genomes. In budding yeast, one of the most striking feature of DSBs is their localization in intergenic intervals containing a transcription promoter, though transcription activity per se is not required [6–8]. However, all transcription promoters do not contain an initiation site. On a large scale, initiation sites are not randomly distributed and are clustered over large chromosomal domains several tens of kb long, often in regions with a high GC content [6,8,9]. In mouse and human, only a small number of contemporaneous recombination hotspots have been identified and characterized. Nevertheless, they do not localize preferentially in promoter regions, contrary to what is observed in budding yeast (see [11]). The genome-wide distribution of historical CO hotspots in human revealed several motifs that are overrepresented in hotspot regions, the most prominent being a 7-mer (CCTCCCT) associated with 11% of the hotspots [26]. Interestingly, the regions with high contemporaneous CO frequency in mouse, defined at the Mb scale, are also enriched for this motif, suggesting that it might correspond to a conserved function across mammals [27]. It should be noted that these short motifs are abundant in the genome, and therefore are not sufficient solely to explain the localization of the hotspots.

A second approach aims to find factors involved in hotspot activity control through the detailed analysis of individual hotspots. In budding and fission yeasts, the presence of open chromatin at initiation sites is important for the formation of DSBs [7,28]. At some hotspots, the binding of transcription factors or chromatin remodeling factors has been shown to be required for DSB formation (reviewed in [6,29]). However, no generalization could be made about the factors regulating recombination initiation. In humans and mice, the activity of several CO hotspots has been shown to vary between individuals (humans) or strains (mice), as shown either by pedigree analysis or by sperm typing. Individual variation in CO rates, up to 75-fold, have been observed for most of the human hotspots that have been analyzed by sperm typing in several individuals [30]. At two of these hotspots, a single nucleotide change at the center of the hotspot appears to be at the origin of a 3- to 6-fold variation in *cis* of the initiation frequency [31,32]. At two others, *MSTM1a* and *MSTM1b,* CO rates are mainly determined by factors other than their own sequence [30]. In mice, a wide range of MHC haplotypes isolated in congenic lines have been instrumental for drawing a picture of haplotype-specific differences in recombination patterns across this region. Some of the hotspots identified in the mouse MHC by pedigree analysis, like the one located in the *Eb* gene, are common to most haplotypes from common laboratory strains. Others, like the *Ea* and *Psmb9* hotspots, are specific to one or a few haplotypes, though a significant rate of CO might remain undetected in other haplotypes due to the insufficient sensitivity of pedigree analyses [18]. The CO rate at these two hotspots appears to be regulated, at least in part, by determinants located outside the interval where exchanges actually occur [33,34].

DNA sequence independent control of meiotic recombination is also illustrated by sex-specific differences in rate and distribution of CO as observed in many species. In human, and to a lesser extent in mouse, overall CO rates are significantly higher in female than in male (4,400 cM versus 2,700 cM in human, reviewed in [35]; 1,800 cM versus 1,400 cM in mouse [27]). Though the succession of regions with high and low CO densities is broadly the same in both sexes, some regions display sex-specific variation. In particular, both in mouse and human, males exhibit higher rates of recombination than females in telomeric regions, while the opposite is true near the centromeres [27,36,37]. The factors at the origin of this sex-specific variation are not known. It has been proposed that the regions subject to parental imprinting may display different recombination rates and therefore that sex-specific epigenetic modifications, such as imprinting, might play a role in the sex-specific distribution of recombination events [38,39]. On a small scale, little information on the sex-specific distribution of CO is available, especially because most information on local CO distribution comes either from the analysis of population diversity (in human), which does not discriminate between sexes, or from sperm typing. Nevertheless, the high concordance observed in a few intervals between the location of the human hotspots predicted from population diversity analyses and the ones detected directly by sperm typing suggests that a large fraction of hotspots are shared by both genders. Therefore, sex-specific differences in CO distribution might be explained by differences in the activity level of the same hotspots rather than by the presence of male- and female-specific hotspots [21,22,24]. Consistent with this view, two human hotspots (*TAP2* and β-*globin*), which have been well characterized by sperm typing, have been shown also to have CO activity in female meiosis [16,40–43]. At the *TAP2* hotspot, available data suggest that the CO frequency might be on average 20- to 40-fold higher in female than in male meiosis [41]. Similarly, there is no evidence for sex-specific activity at the few mouse CO hotspots that have been analyzed in detail by pedigree, with the exception of the hotspot located near the 3′ end of the *Psmb9* gene (previously *Lmp2*) in the class II region of mouse MHC, defined as the *Psmb9* hotspot [19,23,44,45].

Several unique properties of the *Psmb9* hotspot indicate that this region is an interesting model for studying the control of meiotic recombination and understanding the mechanisms involved (e.g., initiation and DSB repair). The formation of a high rate of CO at *Psmb9* requires the presence of either the *wm7* MHC haplotype, derived from *Mus musculus molossinus*, or the *CAS3* MHC haplotype, derived from *M. m. castaneus* [46,47]. The hotspot appears to be female specific in mice carrying the *wm7* haplotype but is active in both sexes carrying the *CAS3* haplotype [46]. However, recombinant mice carrying a shorter *wm7* fragment display a high CO rate in both sexes, demonstrating that the genetic element responsible for the lower CO rate in males is physically distinct from the element at the origin of the high CO rate at this hotspot [33]. To get a better description of the recombination events at the *Psmb9* hotspot and gain insights into the factors controlling its activity, we adapted a PCR-based method recently developed for the direct molecular detection and analysis of both CO and NCO recombination products [13,14]. We extended this method for the detection, quantification, and analysis of recombination products in both male and female germ lines. This method, which provides a sensitivity several orders of magnitude higher than pedigree analysis, allowed us to measure the recombination rate in hybrids in which no recombination can be detected from pedigrees, and therefore to address the questions of both the strain and sex-specificities of recombination activity at *Psmb9* hotspot. Moreover, the analysis of the distribution of exchange points among CO allowed us to infer initiation activity on each homolog within hybrid strains and thus to reveal a complex regulation of recombination initiation at *Psmb9*, involving the interaction of elements acting in *cis* and in *trans*.

## Results

### Mapping of the *wm7* Fragment among Mouse Recombinant Lines

**Figure 1:**
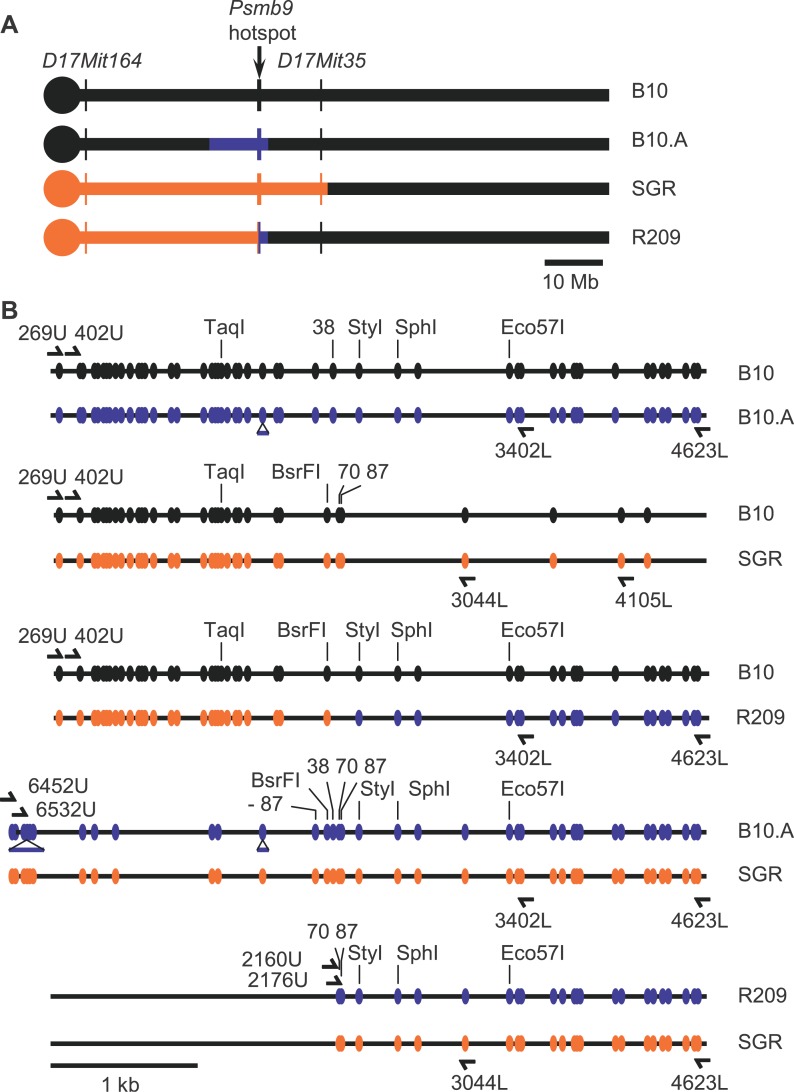
Maps of the Chromosome 17 and the *Psmb9* Hotspot in the Different Strains (A) Origin of the Chromosome 17 fragments in the different parent lines is presented: black, B10; blue, A (haplotype *a*); orange, SGR (haplotype *wm7*). (B) Map of the polymorphisms at the heterozygous state along the *Psmb9* hotspot in the various hybrids is shown. The markers *−87*, *38*, *70*, *87*, *Sty*I, and *Sph*I are 87 bp to the left, and 38, 70, 87, 213, and 474 bp to the right of the *Bsr*FI marker, respectively.

The hybrids used here for analyzing recombination at *Psmb9* have been obtained by crosses between lines that were all congenic to C57BL/10 (abbreviated as B10). The CO hotspot located near the 3′ end of *Psmb9* was identified first in hybrids between a laboratory strain and the strain B10.MOL-SGR(*H-2^wm7^*) (abbreviated as SGR). This strain contains a fragment of Chromosome 17 covering the MHC derived from a wild mouse *M. m. molossinus* (*wm7* haplotype). The *wm7* fragment covers an interval including *H-2K* and *H-2D* in the MHC, but its extent outside this 300-kb region had not been determined [46,48]. We used microsatellite markers to map this *wm7* fragment. It extends well beyond the MHC, covering approximately the proximal half of Chromosome 17 (Figure 1A; Table S1). The most proximal and distal markers to be included are *D17Mit164* (4.1 cM), the closest marker to the centromere analyzed, and *D17Mit35* (23.50 cM), respectively.

The lines used for the various crosses analyzed in the present study were B10, B10.A, SGR, and B10.A(R209) (abbreviated as R209), which differed from each other by their MHC haplotype (Figure 1A). B10.A contains a fragment derived from strain A. SGR contains the fragment of *wm7* haplotype described above. R209 is a recombinant line issued from a CO between SGR and B10.A at the *Psmb9* hotspot [46]. The interval proximal to the *Psmb9* hotspot is identical to that of SGR, while the distal side is identical to that of B10.A. The breakpoint occurred at the center of the hotspot, between markers 38 and 70 (Figure 1B). Thereafter, we designated each Chromosome 17 in hybrids resulting from the cross between two strains by the name of the strain that it comes from. The genetic analysis performed by Shiroishi et al. [33] led to two major conclusions: First, a high frequency of CO (1%–2%) is found at the *Psmb9* hotspot in hybrids carrying the *wm7* haplotype (B10 × SGR, B10.A × SGR, and B10 × R209); second, the high CO activity is specific to female meiosis in hybrids with SGR, but present in both sexes in hybrids with R209.

### Frequencies of Recombination Events at the *Psmb9* Hotspot in Sperm and Oocytes

To get precise evaluation and comparison of the *Psmb9* hotspot activity in both male and female meioses in these various hybrids, we measured CO by direct detection of recombinant molecules in sperm and ovaries. In addition, we set to determine whether the variation in CO rates is specific to the CO pathway or not, by measuring the frequency of NCO products as well. Our previous analyses allowed us to determine the interval where most CO occurred and to define the region of initiation where high frequencies of NCO could be detected [13,14].

First, no CO was detected among 8 × 10^5^ sperm in a hybrid without the *wm7* haplotype (B10 × B10.A), demonstrating that the rate of CO in the 3-kb interval covered by our assay is lower than the genome average of 0.5 cM/Mb (Table 1). In contrast, COs were detected with high rates in both male and female germ lines of every hybrid containing the *wm7* haplotype (0.3%–2.0%; Table 1). Therefore, the presence of the *wm7* haplotype increases CO frequencies at *Psmb9* by 2,000-fold or more. In oocytes, CO frequencies were 2.0% ± 0.4% in B10 × R209, and 1.1%–1.2% ± 0.3% in B10 × SGR and B10.A × SGR. These values are consistent with previous genetic data (1.3%–5.4% and 1.1%–3.3% for R209 × B10 and SGR × B10 or SGR × B10.A, respectively [33]) and thus validate our method for measuring recombination frequencies in oocytes. In hybrids carrying the SGR chromosome (B10.A × SGR and B10 × SGR), the CO frequency was about 4-fold lower in male than in female meiosis (Table 1).

**Table 1:**
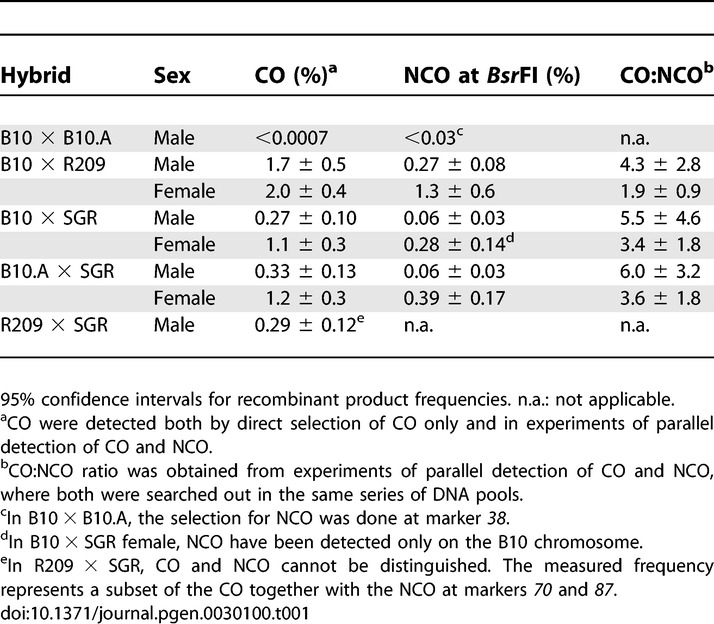
CO and NCO Frequencies at the *Psmb9* Hotspot in Various Hybrids

NCO frequencies were measured at the *Bsr*FI polymorphic site, located close to the center of the hotspot, in most hybrids or at a nearby marker (marker 38) in the B10 × B10.A hybrid, which is homozygous at *Bsr*FI (Figure 1B). As for CO, we have not detected any NCO product in B10 × B10.A sperm DNA (among 22,000 genomes), therefore indicating the absence of any recombination activity. In all other hybrids (B10 × R209, B10 × SGR, and B10.A × SGR), NCO were detected at frequencies varying from 0.06% to 1.3% (Table 1). This variation parallels that observed for CO. Indeed, like CO, the highest rates of NCO were observed in B10 × R209 males and females and in females of B10 × SGR and B10.A × SGR (0.27%–1.3%). In addition, lower, but still detectable, NCO rates were observed in males of these two last hybrids (0.06%). The comparison between CO and NCO frequencies could also be more accurately evaluated by measuring both events in parallel on the same DNA pools, thus allowing a direct determination of the CO:NCO ratio. These ratios are similar between the three hybrids (Table 1, column CO:NCO).

In every hybrid where the activity of the *Psmb9* hotspot has been detected, the *wm7* haplotype was at the heterozygous state in the interval from the centromere to *Psmb9*. Whether the presence of the *wm7* haplotype at the homozygous state in this interval would also be able to induce meiotic recombination at the *Psmb9* hotspot was unknown. To answer this question, we measured the recombination frequency in sperm from an R209 × SGR hybrid, which carries the *wm7* haplotype in the interval from the centromere to *Psmb9* on both homologs (Figure 1A). This hybrid is homozygous for half the interval where exchanges occur, and therefore only a fraction of CO could be detected, those with an exchange point distal to marker *87* (Figure 1B). In addition, the primers cannot discriminate between CO and NCO products with coconversion of markers *70* and *87*, which are only 17 bp apart from each other. As in other hybrids containing either R209 or SGR chromosome, we detected a high level of recombination in this R209 × SGR (0.29% ± 0.12%; Table 1). This demonstrates that the enhancement of recombination at *Psmb9* is an intrinsic property of the *wm7* haplotype, independent from the heterozygosity in the region to the left of marker *70*.

### Control in *Cis* and in *Trans* of Recombination Initiation

**Figure 2:**
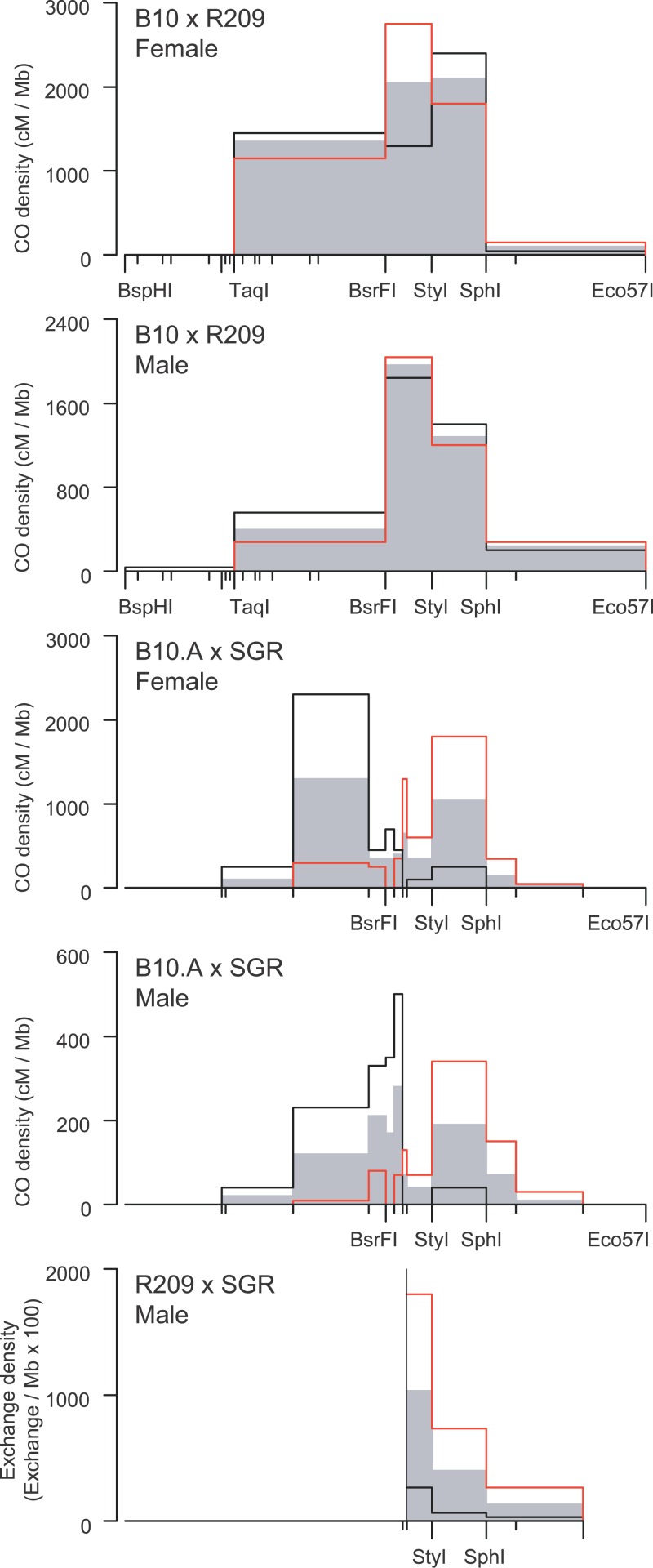
CO Breakpoint Distribution along the *Psmb9* Hotspot CO densities are calculated by dividing the frequency of CO in each interval by its length. CO1 and CO2 represent exchanges in both orientations: black line, CO1 (orientation B10-R209, B10.A-SGR, and R209-SGR, respectively); red line, CO2 (orientation R209-B10, SGR-B10.A, and SGR-R209, respectively); gray area, orientation-averaged CO. Number of events: B10 × R209, female, 112 CO1 and 76 CO2, male, 108 CO1 and 58 CO2; B10.A × SGR, female, 45 CO1 and 69 CO2, male, 62 CO1 and 62 CO2; R209 × SGR, male, 21 CO1 and 73 CO2.

We examined in detail the distribution of CO exchange points in B10 × R209 and B10.A × SGR. Most of the main properties of the distribution of CO along the hotspot are conserved between these hybrids in both sexes (Figure 2): Exchanges are distributed over 2.5 kb (90% of exchanges over 1.2 kb), centered on the 210-bp *Bsr*FI-*Sty*I interval, with densities decreasing progressively on both sides of the hotspot. However, B10 × R209 and B10.A × SGR displayed striking differences in the distribution of exchanges. In B10 × R209, the highest density of exchanges lies at the center of the hotspot, with a gradient of decreasing densities on both sides. In contrast, in B10.A × SGR, the region at the center of the hotspot (interval *70*-*Sty*I) displayed a 2- to 5-fold lower exchange density than the surrounding intervals (Figure 2). Mechanisms that could explain these observations are discussed below.

**Figure 3:**
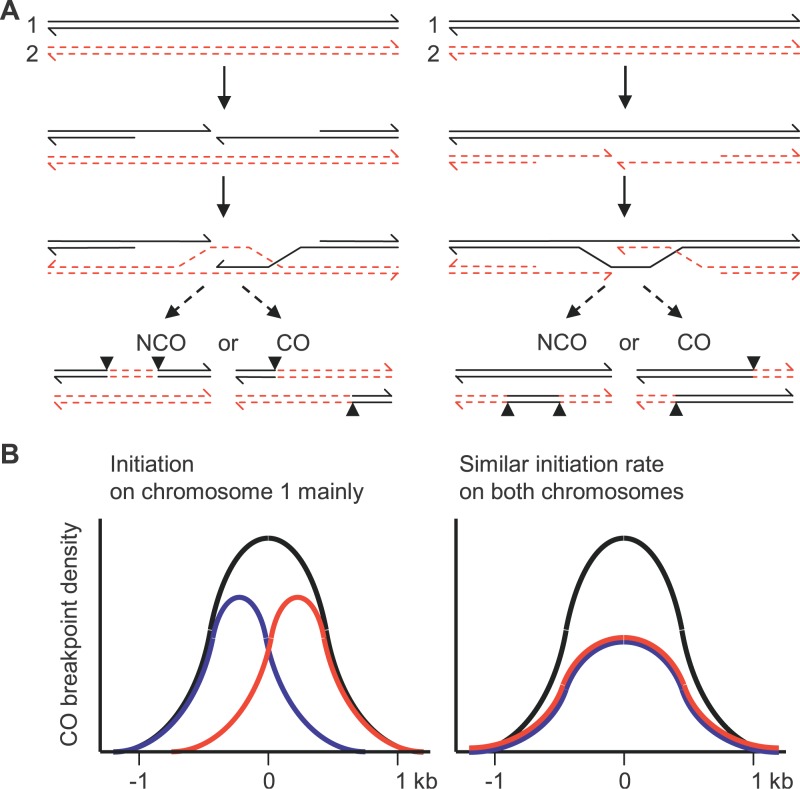
Distribution of Exchange Points at an Initiation Site (A) A model for meiotic recombination is shown. The region surrounding the initiating DSB, between the breakpoints (arrowheads), is converted. The molecule having suffered the initiating DSB is the recipient of genetic information [2,3]. The CO exchange breakpoints are shifted in opposite directions, depending on which is the initiating chromosome (left panel, Chromosome 1 and right panel, Chromosome 2). (B) The expected distribution of CO exchange breakpoints at an initiation site is shown: blue, Chromosome 1 to Chromosome 2 orientation; red, Chromosome 2 to Chromosome 1 orientation; black, sum of exchanges in both orientations. On the left panel initiation is mainly on Chromosome 1. On the right panel initiation on both chromosomes is with similar frequency.

Following the current models for meiotic recombination, the sequences surrounding the initiating DSBs are converted in both NCO and CO products (Figure 3). Therefore, the initiation activity can be evaluated on one and the other homologous chromosome in a given hybrid by measuring the conversion frequencies in the corresponding directions. For NCO, this is achieved by measuring the NCO frequency on each homolog. For CO, the conversion tracts cannot be detected directly. However, we took advantage of the fact that the exchange points of the two reciprocal products of a CO event are located on opposite sides of the conversion tract (Figure 3). Consequently, if initiation is more frequent on one homolog than on the other, the distribution of exchange points for CO products in each orientation are expected to be shifted relatively to each other in a direction depending upon which homolog initiates more frequently (Figure 3 and [31]). Associated with this shift, a transmission bias favoring the allele of the chromosome with a lower initiation rate is expected for the markers closest to the center of the hotspot. Conversely, if initiation occurs at equivalent frequencies on both homologs, exchange points for CO products in both orientations are expected to display a similar distribution, and no transmission bias should be observed.

We first compared NCO frequencies on one and the other homolog in B10 × R209; they were undistinguishable (Table 2). Then, we examined the distribution of reciprocal exchanges in the same hybrid. Distributions of exchanges in one orientation (i.e., B10–R209) and the other (R209–B10) were similar (χ^2^, *p* > 0.2 in both sexes), each of them following the overall distribution of CO (Figure 2). Together, these observations suggested that recombination events giving rise to both NCO and CO are initiated at the same frequency on R209 and B10 Chromosomes. The presence of events initiated on the B10 Chromosome demonstrated that initiation at the *Psmb9* hotspot is activated in *trans* by a *wm7*-specific element.

**Table 2:**
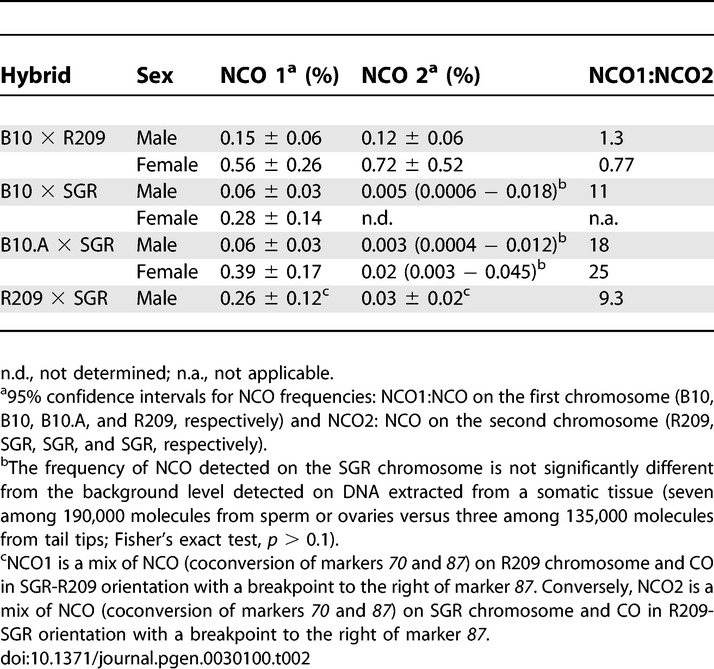
NCO Frequencies in Both Orientations at *Psmb9* Hotspot

**Figure 4:**
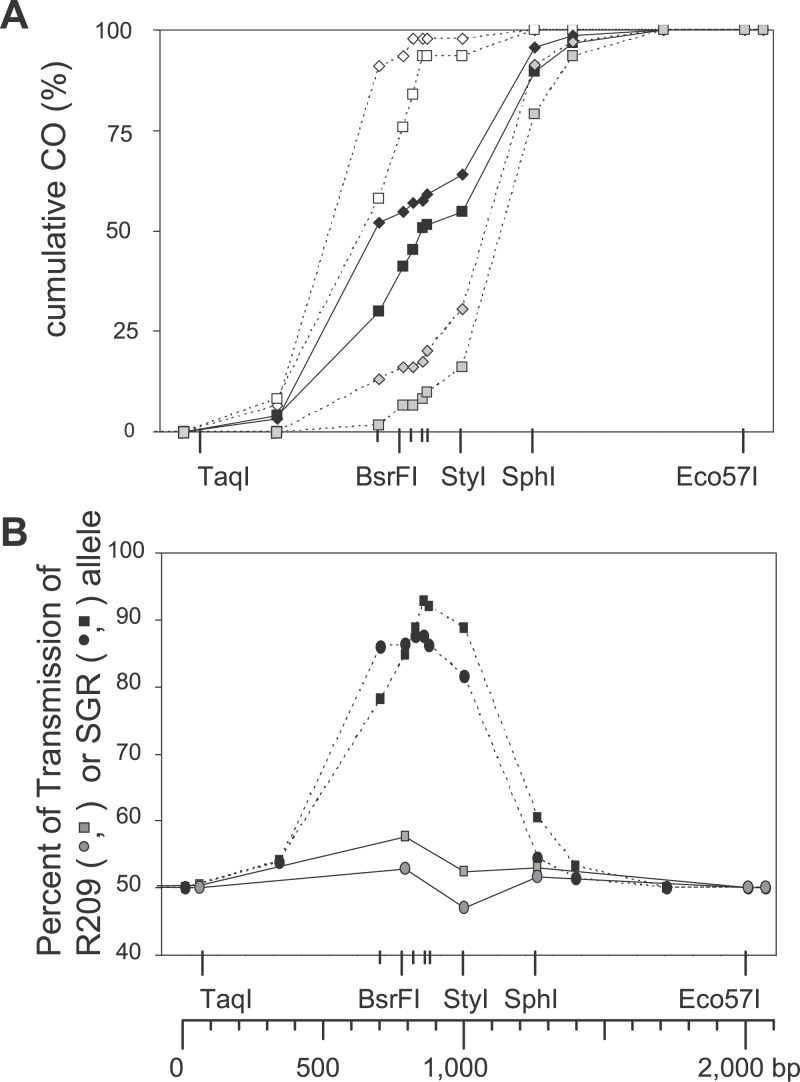
Cumulative CO Distribution and Transmission Bias in Males and Females (A) CO distribution in B10.A × SGR is presented: open diamonds, female, orientation B10.A-SGR; open squares, male, orientation B10.A-SGR; gray diamonds, female, orientation SGR-B10.A; gray squares, male, orientation SGR-B10.A; black diamonds, female, both orientations; black squares, male, both orientations. (B) Transmission bias among CO products is presented; transmission frequency of the *wm7* allele in B10 × R209 and B10.A × SGR. Gray circles and squares, B10 × R209, female and male, respectively; black circles and squares, B10.A x SGR, female and male, respectively.

In contrast, in B10.A × SGR, NCO were at least ten times more frequent on B10.A chromosome than on the SGR chromosome (Table 2). Moreover, the distribution of exchanges in each orientation was different, with B10.A-SGR exchanges being displaced to the left and SGR-B10.A exchanges to the right (Figures 2 and 4A). As explained above, an explanation for these distributions is that most COs result from events initiated on the B10.A chromosome. This asymmetry, observed in both sexes, is accompanied by the overtransmission of the SGR alleles among exchange molecules for markers located close to the center of the hot spot, reaching 93% in male and 88% in female for the marker *70* (Figure 4B). Similar results were obtained in the B10 × SGR hybrid (Table 2 and unpublished data). Together, these observations suggested that initiation is at least ten times less frequent on the SGR chromosome than on a non-SGR chromosome (i.e., B10.A or B10). In addition to giving insight on the initiating chromosome, the extent of the shift between the distributions of CO in each orientation also provides a minimal estimate of the average length of the associated conversion tracts. The curves of cumulative frequencies for B10.A × SGR hybrids indicated that conversion tracts associated with CO were approximately 500 bp long, in both sexes (Figure 4).

### Different Distribution of CO Events in Both Sexes

**Figure 5:**
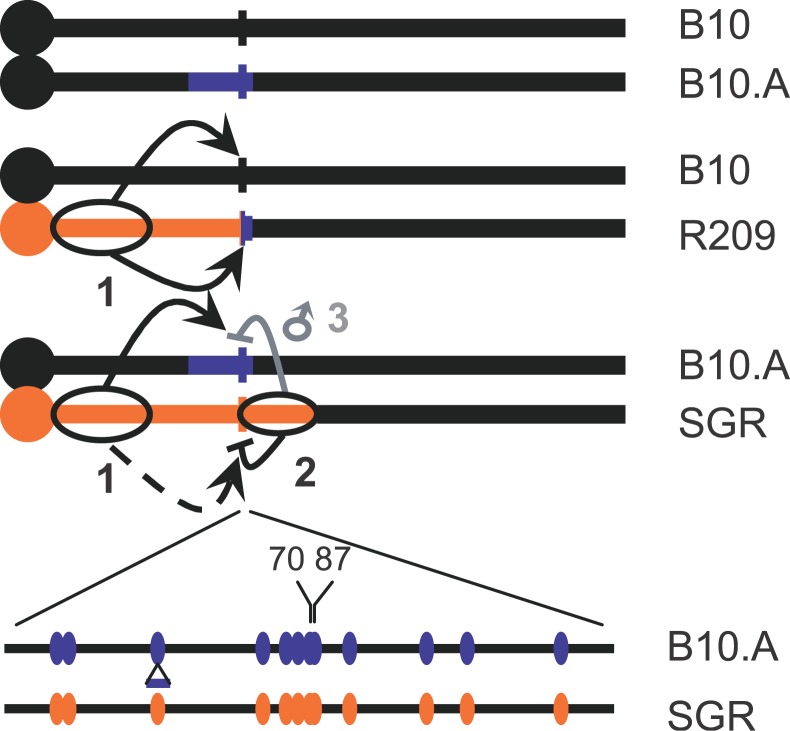
Genetic Control in *Cis* and in *Trans* of Meiotic Recombination at the *Psmb9* Hotspot 1, *wm7*-specific *trans*-acting activator; 2, *cis*-acting repressor specific for SGR chromosome; 3, sex-specific control in *trans* of the recombination rate.

The numerous recombinant products recovered by our method allowed us to do a fine-scale analysis of the distributions of CO between male and female in the B10.A × SGR hybrid, which has the highest density of polymorphisms around the center of the hotspot (Figure 1). The curves of cumulative frequencies revealed a shift of about 100 bp to the left for the distribution of exchanges in female compared to male (Figures 4A and 5). In this hybrid, in which the distributions of CO in each orientation were different, this 100-bp shift was observed for both orientations (Figure 4A). A similar shift of CO distribution in female relatively to male was observed in B10 × R209 (Figure 2). These differences between male and female CO distributions were statistically significant in both hybrids (χ^2^, *p* < 0.05).

## Discussion

Our analysis leads to two main conclusions on the regulation of recombination initiation at the *Psmb9* hotspot. First, the presence of a *wm7*-specific element induces recombination initiation at the *Psmb9* hotspot in *cis* and in *trans*, both on the *wm7* chromosome and on its non-*wm7* homolog (element 1 on Figure 5). Second, recombination initiation is repressed on the SGR chromosome, suggesting a *cis*-acting regulation observed in both sexes and in all hybrids carrying the SGR chromosome (element 2 on Figure 5). This interplay between activation in *trans* and repression in *cis* leads to the surprising observation that in B10 × SGR and B10.A × SGR, most recombination at the *Psmb9* hotspot initiates on the non-*wm7* chromosome, although it is dependent on the *wm7* haplotype.

### Variations in CO Rates at *Psmb9* Result Mostly from Variations in Initiation Rates

In principle, a variation in CO frequency could result either from a change of the DSB frequency or from a change of the proportion of DSBs repaired towards CO rather than NCO. The parallel analysis of CO and NCO in the various hybrids and sexes analyzed showed that the variations of CO and NCO rates were largely correlated. In particular, the 10-fold reduction of frequencies of CO initiated on the SGR chromosome is correlated with a similar reduction of NCO frequencies (see Results and Table 2). These observations indicate that most of the variation in CO frequencies measured at *Psmb9* hotspot results from the variation in the initiation rate.

### A *Trans*-Acting Locus-Specific Enhancer of Meiotic Recombination

The presence of the *wm7* haplotype is required for the formation of recombinant products at the *Psmb9* hotspot. Indeed, neither CO nor NCO has been detected in sperm from the B10 × B10.A hybrid, which lacks the *wm7* haplotype. The absence of CO in 8 × 10^5^ sperm from the B10 × B10.A hybrid indicates that the density of residual COs in the 3-kb interval amplified in our assay, if there are any, is less than 0.2 cM/Mb. Sperm-typing analyses of one mouse hotspot *(Eb)* and 16 human hotspots evidenced a moderate variation in CO frequencies, up to 76-fold, but there is only one documented example of a mammalian CO hotspot that is active in some individuals and inactive in others [30,49]. Therefore, the increase by more than 2,000-fold of the CO frequency at *Psmb9* hotspot induced by the presence of the *wm7* haplotype is by far the largest genetically controlled variation at a mammalian hotspot to date (Table 1, compare B10 × R209 with B10 × B10.A).

The *wm7* haplotype-specific element that activates recombination at *Psmb9* has several unusual properties, summarized in Figure 5 (element 1). First, the mapping of CO breakpoints (Figure 2) together with the detection of NCO on both homologous chromosomes (Table 2) demonstrate that recombination initiation is activated in *trans*, both on the *wm7* chromosome and on the homologous, non-*wm7* chromosome. Second, the hotspot activator is physically distinct from the interval where exchanges actually occur, as noted by Shiroishi et al. [33]. By producing and analyzing an additional recombinant line, we confirmed that this element is located outside the hotspot (F. Baudat and B. de Massy, unpublished data). Third, the following observations indicate that the increase of recombination activity is specific to one *(Psmb9)* or a limited number of loci. There is no global genome-wide or even chromosome-wide increase of CO, as evidenced by the normal number of Mlh1 foci on pachytene spermatocytes (C. Grey and B. de Massy, unpublished data). Only little differences outside the *Psmb9* hotspot have been found by pedigree analysis of the whole MHC between the hybrids that carry the *wm7* haplotype and the ones that do not [46]. Moreover, a classical genetic mapping study failed to show any increase of the genetic length along the proximal half of Chromosome 17 in B10 × R209 hybrids, except for the interval containing *Psmb9* (B. de Massy, unpublished data).

The finding of a haplotype-specific element activating recombination at a specific locus raises the question of what is the mechanism involved in this process. The size of the recombination activating element is not known. The specificity of the target (the *Psmb9* hotspot) suggests that a specific interaction, either direct or indirect, between the recombination activator and the locus of the hotspot is involved. Several hypotheses could be proposed. One is that the activator is a gene, coding for a factor interacting with the *Psmb9* hotspot. There are several examples in which factors, such as transcription or chromatin-modifying factors, are required for detectable levels of recombination initiation at specific yeast hotspots (for example, see [50,51]). An alternative mechanism involves a long-distance interaction between the activator (of *wm7* haplotype) and the hotspot. Cases of long-distance locus-to-locus associations have already been reported, such as between *Igf2/H19* and *Wsb1/Nf1*, or between the H enhancer and one olfactory receptor gene promoter in olfactory sensory neurons [52,53].

### A *Cis*-Acting Repressing Element

A gene conversion bias favoring the alleles from the SGR chromosome is observed for both NCO and CO products (Figure 4B; Table 2). This could in theory be explained either by a bias in initiation or a bias during the mismatch repair of the heteroduplex DNA formed in recombination intermediates. However, we consider the last hypothesis is unlikely, because biased mismatch repair leading to restauration would be expected to increase the frequency of exchange points near initiation, a situation that we did not observe (Figure 2). Therefore, our data show that the rate of initiation is at least 10-fold lower on the SGR chromosome than on the homologous non-SGR chromosome. This suggests strongly that a *cis*-acting repressing element is present on the SGR chromosome. One could note that the repression of initiation was identical whether the SGR chromosome has been transmitted to the hybrid by its mother or father, eliminating the hypothesis that this *cis*-effect results from a mark dependent upon the sex of the parent having transmitted the chromosome (unpublished data). The element at the origin of the repression of recombination initiation on the SGR chromosome should be located in the interval differing between SGR and R209, which includes the marker *70* at the center of the hotspot and extends up to at least *D17Mit35,* 11 Mb centromere distal to *Psmb9,* (Figure 1; Table S1). The two SNPs, *70* and *87,* being located in the predicted region of initiation (Figure 1), are interesting candidates. There are already two documented cases (the human hotspots *DNA2* and *NID1*) where a particular allele for a single SNP located at the hotspot center correlates with the repression in *cis* of recombination initiation [31,32].

### Sex-Specific Regulation

The activity of the *Psmb9* hotspot, evaluated by pedigree analysis, has been reported previously to be female specific in hybrids carrying the SGR chromosome [33]. Our more sensitive assay demonstrates that this is not the case, but reveals a moderate decrease of 4- to 7-fold in male recombination rate compared to female (Table 1). Interestingly, this male-specific effect of the SGR chromosome involves recombination products resulting from initiation on the non-SGR chromosome, indicating a regulation in *trans*. Like the *cis* effect described above, this SGR-specific regulation could be due to markers *70* and/or *87* or other SGR-specific elements in the interval from marker *70* to *D17Mit35*. The lowering of recombination rate in males could result either from an effect on the level of initiation or on the processing of recombination intermediates. Two types of sex-specific mechanisms could be invoked, either related to the presence of a diffusible factor or to a specific interaction between homologs. Consistent with the last hypothesis, interactions in *trans* between homologous chromosomes have been shown to modulate the frequency of recombination at S. cerevisiae hotspots [54,55]. Perhaps reflecting a similar process, a sperm typing study at the mouse *Eb* hotspot showed that the rate of CO resulting from initiation on the chromosome of a particular haplotype (haplotype *s*) is modified depending on the haplotype of the homologous chromosome [49]. Whatever the molecular mechanism is, this effect might be another manifestation of the mechanism that is responsible for the repression in *cis* of recombination initiated on the SGR chromosome, described above.

### Subtle Variation in CO Distribution at the *Psmb9* Hotspot

In addition to the determinants controlling the rate of recombination, strain- and sex-specific differences have also been detected in the distribution of recombination products along the sequence of the hotspot.

The overall distribution of CO at the *Psmb9* hotspot in B10 × R209 is similar to the other mouse and human hotspots that have been characterized so far. In particular, the CO density peaks at the center of the hotspot and decreases progressively on both sides over a few hundreds bases [11–13]. Quite differently, in B10.A × SGR, the CO density is higher on both sides of the hotspot than in a 140–300-bp central interval where it is about 4-fold lower (Figure 2). However, NCO frequencies are higher at markers located in the *Bsr*FI-*Sty*I interval (markers *38*, *70,* and *87*) than at *Bsr*FI and *Sty*I, suggesting that most of the initiation occurs inside this interval (F. Baudat and B. de Massy, unpublished data). Therefore, the observation that most CO exchanges points are outside the *Bsr*FI-*Sty*I interval suggests that the gene conversion tracts associated with CO are bidirectional, extending on both sides of the initiating DSBs with a minimal length of about 300 bp overall. We found that two types of hypotheses (not mutually exclusive) could explain this difference between the B10.A × SGR and B10 × R209 hybrids. With the first hypothesis, initiating DSBs would be distributed on a shorter interval in B10.A × SGR than in B10 × R209. Alternatively, a difference in the processing of recombination intermediates might result in longer gene conversion tracts in B10.A × SGR than in B10 × R209. This could be due to a higher density of heterozygosity near the initiation site. A similar situation has been observed in one individual at the human *DPA1* hotspot, where the density of CO breakpoint was lower at the center of the hotspot, in an interval with a particularly high density of heterozygous SNPs, than in the neighboring intervals on both sides (Figure 4A in [21]).

In addition, the shift by about 100 bp to the left in female compared to male, observed in both B10.A × SGR and B10 × R209, suggests that the location of the initiating DSBs can vary independently from the DNA sequence (Figures 2 and 4A). In B10.A × SGR this shift is observed independently for exchanges in each orientation and therefore involves both sides of the conversion tracts (Figure 4A). We therefore consider the possibility of a difference in the processing of recombination intermediates unlikely, as it would require the simultaneous shortening and lengthening of conversion tracts on each side of initiation. Therefore, the simplest explanation is a difference in the location of DSBs, which would then be predicted to be shifted to the left by about 100 bp in female meiosis. A possibility to account for a difference in the localization of initiating lesions is a difference in chromatin organization between spermatocytes and oocytes, such that the accessible region to the recombination initiation machinery is not exactly at the same position in both sexes.

### Implications for Hotspot Dynamics

The so-called hotspot paradox refers to the fact that hotspots are maintained in genomes despite the evolutionary force that tends to eliminate them [56–59]. Indeed, in the current models for meiotic recombination, the region surrounding the initiating DSB is converted into the allele of the noninitiating chromosome [2,3]. Therefore, if the rate of DSB formation at a given hotspot is controlled in *cis* by elements localized in the converted interval, alleles displaying a low initiation rate are predicted to replace progressively the “hotter” alleles. This meiotic drive is expected to act against the introduction of new hotspots in the population, as well as to favor the fixation of the less active alleles and eventually the extinction of existing hotspot [56–59]. The continuous presence of recombination hotspots in genomes might therefore be ensured by a mechanism of turnover, which would compensate for the extinction of some hotspots with the birth of new ones [22]. Coop and Myers proposed that the newly arising hotspots that escape a premature elimination do not initially experience this meiotic drive [59]. This could be achieved if the elements responsible for hotspot activity are localized outside the frequently converted region and/or enhance the initiation of recombination in *trans*. At established hotspots, in contrast, *cis*-acting local alleles repressing recombination are expected to spread over the population, owing to the mechanism of meiotic drive described above.

The regulation of recombination at the *Psmb9* hotspot displays properties consistent with this model. First, the *wm7*-specific genetic element activating recombination is localized outside the hotspot and acts in *trans* (Element 1 on Figure 5). Therefore this element could be responsible for the existence of the *Psmb9* hotspot without being affected by the mechanism of meiotic drive described above. This might also be exemplified at the human *MSTM1a* hotspot, of which the activity is controlled by factors other than its own sequence [22]. Second, as discussed above, the repression in *cis* of recombination initiation on the SGR chromosome might be controlled by one or two SNPs localized at the center of the hotspot (markers *70* and *87*). The putative repressive alleles (from the SGR chromosome) represent the derived state of these two markers, which is consistent with the idea that the spreading of such repressing alleles in a population is favored in the presence of the active hotspot (comparison with the sequence of Rattus norvegicus from Ensembl, RGSC3.4 of December 2004, http://www.ensembl.org/Rattus_norvegicus/index.html). Interestingly, at *DNA2* and *NID1* human hotspots, the repression also occurs specifically on chromosomes carrying the derived allele for a SNP located at the center of the hotspot [32].

Our data and other studies on recombination hotspots thus reveal the various levels of hotspot control that could be either DNA sequence dependent or not and that are able to act locally or at distance. These controls are indeed consistent with the observation that a communication has to operate somehow along each chromosome arm to produce, among other things, at least one CO per chromosome arm. Given the current explosion of hotspots identified in mammals [60], understanding their control elements are significant challenges for the future.

## Materials and Methods

### Mouse strains.

The mouse lines used in this study were C57BL/10JCrl (purchased from Charles River Laboratories, http://www.criver.com), B10.A (purchased from The Jackson Laboratory, http://www.jax.org), and B10.MOL-SGR and B10.A(R209) (from T. Shiroishi, National Institute of Genetics, Mishima, Japan). B10.MOL-SGR was established by repeated backcrosses (13 generations) to the C57BL/10J background [61].

### Detection of CO and NCO molecules.

The strategy of the PCR-based method developed for the direct molecular detection and analysis of recombination products in male and female germ lines has been described in detail in Guillon et al. (Figure S1) [14]. The primers used for the detection of CO and NCO in the various hybrids are listed in Tables S2 and S3. The method was adapted for estimating the absolute frequencies of recombination products in oocytes. A cell suspension was obtained by EDTA collection of the pooled ovaries from a litter of new-born mice, according to [62], modified as follows. Ovaries were incubated in 1 mM EDTA in PBS at room temperature for 30 min, after which cells were released by pricking the gonads with fine needles in 0.05% BSA in PBS. The cells were collected in 500 μl 0.05% BSA in PBS. The proportion of oocytes was determined on a 25-μl aliquot of each cell suspension by immunofluorescence with antibodies recognizing germ cell-specific antigens, GCNA1 (gift from G. Enders, University of Kansas, Kansas, United States of America) and SYCP3 (prepared in guinea-pig against a mouse SYCP3 oligopeptide) (Figure S2). DNA was extracted from the remainder of each cell suspension and used to detect recombinant products. Estimates of recombinant frequencies were eventually corrected for the proportion of oocytes in each suspension, assuming that all detected recombinant events occurred in oocytes and that the genomic content of oocytes is 4n versus 2n for most somatic cells. The primers used in the various hybrids are shown in Figure 1 and Table S2.

Frequencies of recombination events were given by the ratio between the mean number of events per pool and the number of amplifiable genomes, both calculated according to the Poisson law. For estimating both the concentration of amplifiable molecules and the number of recombinant products, dilutions were chosen such that the proportion of negative pools was comprised between 0.2 and 0.8. The standard deviation was estimated using the normal approximation of the Poisson distribution, and 95% confidence intervals were calculated as the estimate of the recombination frequency plus or minus 1.96 standard deviation. For comparing two frequencies, the difference between the frequencies to be compared was estimated, with its standard deviation. The statistical significance of the observed difference was then evaluated (at the 5% significance level) by examining if the value of 0 was enclosed in the interval made by the estimate of the difference plus or minus 1.96 standard deviation [63]. When the frequency of recombinant products was too low, or when no recombinant product was detected, the 95% confidence interval was calculated using the Poisson approximation for the binomial distribution of the recombinant product number over the estimate of the total number of amplifiable molecules.

CO were detected either by a direct selection involving two rounds of allele-specific PCR or by the assay of parallel detection of CO and NCO described in [14]. The CO:NCO ratio was the ratio between the mean number of CO and the mean number of NCO estimated in the same series of pools in the parallel assay.

For every hybrid, DNA extracts from at least two mice (males) or two litters (females) have been analyzed independently, with the exception of B10 × SGR males for which only one individual has been analyzed. CO frequencies measured in different individuals of identical genotype were not significantly different (*p* > 0.1), with the exception of B10 × SGR females, for which DNA extracts from three litters gave CO frequencies of 0.6 ± 0.2%, 1.1 ± 0.6%, and 1.6 ± 0.8%, the two extreme values being significantly different from each other (0.02 < *p* < 0.05). Nevertheless, all three CO frequencies were significantly higher than the male CO frequency when analyzed separately and were pooled for generating the data presented in this study.

### Mapping of exchanges.

The intervals where exchanges occurred were mapped with restriction site polymorphisms or by sequencing of the secondary PCR products. The number of events detected in each interval was corrected according to the Poisson distribution as described in [64]. The distributions of exchange points were compared by performing a χ^2^ test on the numbers of exchange points in the various intervals.

### Microsatellite marker mapping.

The sequences of the primers used for mapping the markers listed in the Table S1 have been found on the Mouse Genome Informatics site (http://www.informatics.jax.org). The PCR cycling conditions were 94 °C for 10 s, 55 °C for 30 s, and 72 °C for 30 s for 36 cycles.

## Supporting Information

Figure S1 Example of the Parallel Detection of CO and NCO (174 KB PDF)

Figure S2 Detection of Oocytes in an Ovary Cell Suspension (45 KB PDF)

Table S1 Mapping of the Fragment Derived from *M. m. molossinus* in SGR (52 KB DOC)

Table S2 Sequences of the Primers Used in This Study (60 KB DOC)

Table S3 Combinations of Primers Used for the Various PCRs (72 KB DOC)

## Abbreviations

CO: crossover
DSB: double-strand break
MHC: major histocompatibility complex
NCO: gene conversion without associated crossover
R209: B10.A(R209)
SGR: strain B10.MOL-SGR(*H-2^wm7^*)
