# Supplementary material for: Cis- and Trans-Acting Elements Regulate the Mouse Psmb9 Meiotic Recombination Hotspot
Source: PLoS Genet. 2007 Jun 22;3(6):e100. doi: 10.1371/journal.pgen.0030100 (PMC1892046; doi:10.1371/journal.pgen.0030100)
Supplement: Supplementary file 1 [file pgen.0030100.sg001.pdf]

Figure S1

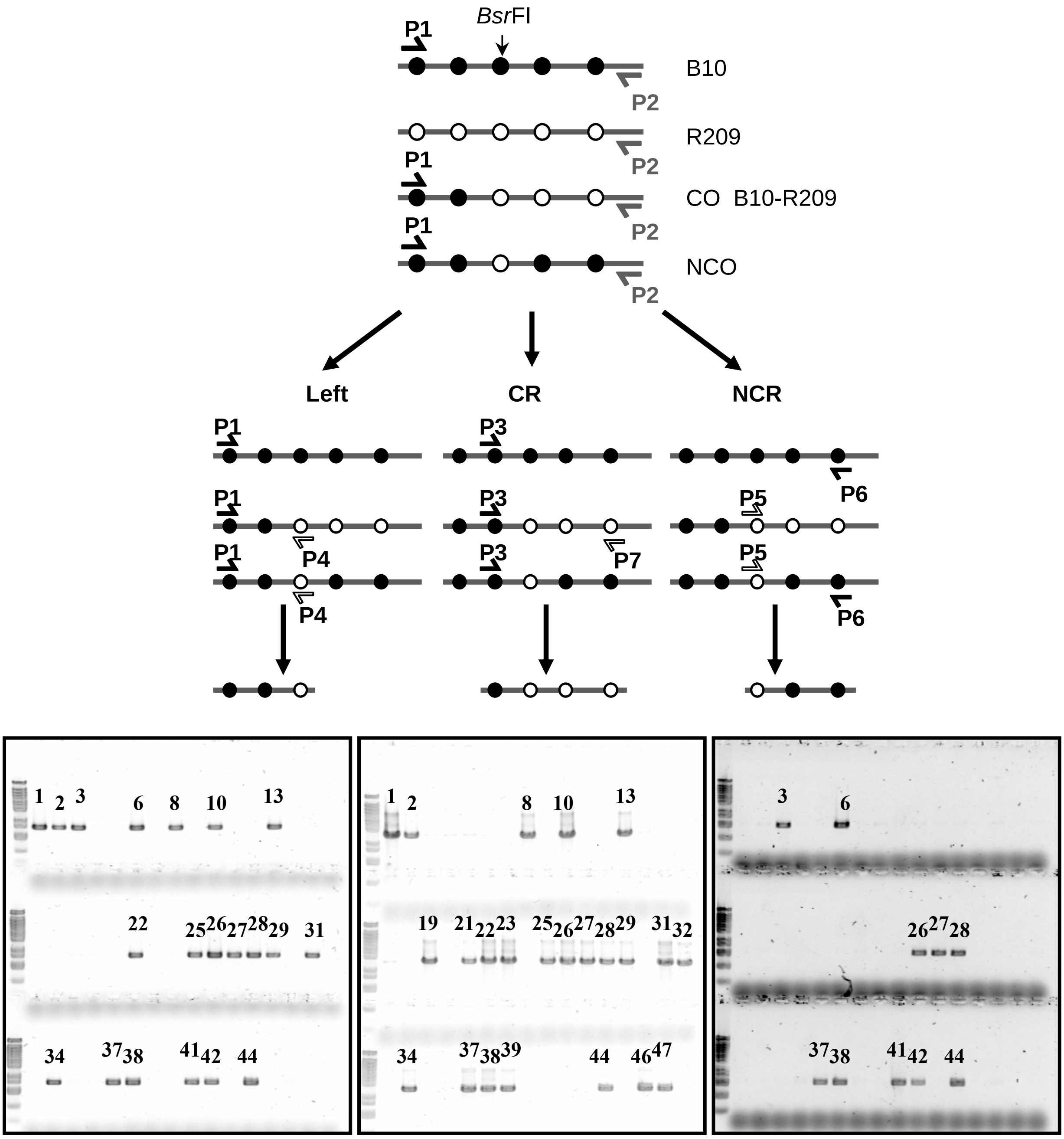

CO (left of BsrFI)
NCO

CO

NCO
