# Supplementary material for: Cis- and Trans-Acting Elements Regulate the Mouse Psmb9 Meiotic Recombination Hotspot
Source: PLoS Genet. 2007 Jun 22;3(6):e100. doi: 10.1371/journal.pgen.0030100 (PMC1892046; doi:10.1371/journal.pgen.0030100)
Supplement: Supplementary file 3 [file pgen.0030100.st001.doc]

# Table S1: Mapping of the fragment derived from M. m. molossinus in B10.MOL-SGR(H-2*^wm7^*).

| Marker name | Position (Mb)  Ensembl release 41 (Oct 2006)  Length of chr. 17: 95.18 Mb | Position (cM)  (MGI 3.51)  Length: 58 cM | Allele present in  B10.MOL-SGR ^a^ |
| --- | --- | --- | --- |
| *D17Mit164* | 3.88 | 4.1 | *wm7* |
| *D17Mit19* | 4.76 | 3.0 | *wm7* |
| *D17Mit143* | 8.28 | 5.0 | *wm7* |
| *D17Mit113* | 11.82 | 6.5 | *wm7* |
| *D17Mit27* | 7.25, 12.99, 13.23 | 7.54 | *wm7* |
| *D17Mit59* | 18.21 | 9.91 | *wm7* |
| *D17Mit197* | 19.83 | 9.65 | *wm7* |
| *D17Mit133* | 24.59 | 10.4 | *wm7* |
| *D17Mit30* | 27.22 | 16.4 | *wm7* |
| *D17Mit198* | 27.39 | 16.0 | *wm7* |
| *D17Mit28* | 33.61 | 18.44 | *wm7* |
| *Psmb9* | 33.79 | 18.59 | *wm7* |
| *D17Mit21* | 33.85 | 18.64 | *wm7* |
| *D17Mit34* | 34.35 | 18.8 | *wm7* |
| *D17Mit176* | 42.20 | 22.5 | *wm7* |
| *D17Mit50* | 44.82 | 23.2 | *wm7* |
| *D17Mit35* | 45.01 | 23.5 | *wm7* |
| *D17Mit200* | 45.60 | 24.2 | *= b* |
| *D17Mit36* | 46.28 | 24.5 | *= b* |
| *D17Mit67* | 48.72 | 25.0 | *= b* |
| *D17Mit70* | 52.16 | 32.7 | *= b* |
| *D17Mit239* | 56.33 | 34.3 | *= b* |
| *D17Mit88* | 56.91 | 29.5 | *= b* |
| *D17Mit20* | 56.91 | 34.3 | *= b* |
| *D17Mit93* | 73.71 | 44.5 | *= b* |

^a^ *wm7*, the allele present in SGR is different from the allele present in B10 for this marker. *= b*, the allele in SGR cannot be distinguished from the allele present in B10.
