# Supplementary material for: Cis- and Trans-Acting Elements Regulate the Mouse Psmb9 Meiotic Recombination Hotspot
Source: PLoS Genet. 2007 Jun 22;3(6):e100. doi: 10.1371/journal.pgen.0030100 (PMC1892046; doi:10.1371/journal.pgen.0030100)
Supplement: Supplementary file 4 [file pgen.0030100.st002.doc]

# Table S2: Sequences of the primers used in this study.

| Name | Sequence | Strain specificity | Marker |
| --- | --- | --- | --- |
| 269U20 | TTAGCTTCTGAGCCATCTGT | B10 |  |
| 269U20b | TCAACTTCTGAGCCGTCTGA | B10.A, SGR, R209 |  |
| 6452U22A | GAAACCCCTTAGTGGATCTATT | B10.A |  |
| 6452U22S | GAAACCCTTTAGTGGATCTACG | SGR |  |
| 402U19 | CTTCCTCTTGCCATTACCG | B10 |  |
| 402U19b | CCTCCTCCTACCATTACCT | B10.A, SGR, R209 |  |
| 6532U20A | AGGGAACTGAACTCCAGTTT | B10.A |  |
| 6534U18S | GGAGCTGAACTCCAGTCC | SGR |  |
| 2092U16b10 | GTTGTGCAAGATGCCT | B10, B10.A | *Bsr*FI |
| 2092U16R209 | GTTGTGCAAGATGCCG | SGR, R209 | *Bsr*FI |
| 2128U18A | ACAGCTTCAAGGGCTCTC | B10.A | marker *38* |
| 2128U18S | ACAGCTTCAAGGGCTCTG | B10, SGR, R209 | marker *38* |
| 2160U18b10 | CACGTGGATCCAGGGAAT | B10, B10.A, R209 | marker *70* |
| 2160U17sgr | ACGTGGATCCAGGGAAC | SGR | marker *70* |
| 2176U17b10 | AGAACTTTGACCATTAC | B10, B10.A, R209 | marker *87* |
| 2176U17sgr | AGAACTTTGACCATTAT | SGR | marker *87* |
| 4623L20 | ATTTGAGAGTGGGAGGGGAT | B10, SGR |  |
| 4623L20b | ATTTGAGAGTGAGAGGGGAC | B10.A, R209 |  |
| 3402L20 | AATAAAGGGCTAGCTGTGCA | B10, SGR |  |
| 3402L20b | AATAAAGGGCTGGCTGTGCC | B10.A, R209 |  |
| 3044L18b10 | GGGTACAAACCATTGCCC | B10, B10.A, R209 |  |
| 3044L19sgr | AGGGTACAAACCATTGCCT | SGR |  |
| 3044L14b10 | ACAAACCATTGCCC | B10, B10.A, R209 |  |
| 3044L15sgr | TACAAACCATTGCCT | SGR |  |
| 2107L16b10 | GACCTCAGGGCACACA | B10, B10.A | *Bsr*FI |
| 2107L16R209 | GACCTCAGGGCACACC | SGR, R209 | *Bsr*FI |
