# Supplementary material for: Cis- and Trans-Acting Elements Regulate the Mouse Psmb9 Meiotic Recombination Hotspot
Source: PLoS Genet. 2007 Jun 22;3(6):e100. doi: 10.1371/journal.pgen.0030100 (PMC1892046; doi:10.1371/journal.pgen.0030100)
Supplement: Supplementary file 5 [file pgen.0030100.st003.doc]

# Table S3: Combinations of primers used for the various PCRs.

| PCR | Hybrid | Orientation | Forward primer | Reverse primer |
| --- | --- | --- | --- | --- |
| 1 | B10 x B10.A | 1 | 269U20 | 4623L20 + 4623L20b |
| 2, CO | B10 x B10.A | 1 | 402U19 | 3402L20b |
| 2, NCO | B10 x B10.A | 1 | 2128U18A | 3402L20 |
| 1 | B10 x B10.A | 2 | 269U20b | 4623L20 + 4623L20b |
| 2, CO | B10 x B10.A | 2 | 402U19b | 3402L20 |
| 2, NCO | B10 x B10.A | 2 | 2128U18S | 3402L20b |
| 1 | B10 x SGR | 1 | 269U20 | 4623L20 |
| 2, CO | B10 x SGR | 1 | 402U19 | 3044L19sgr |
| 2, NCO | B10 x SGR | 1 | 2092U16R209 | 3044L18b10 |
| 2, left | B10 x SGR | 1 | 269U20 | 2107L16R209 |
| 1, CO only * | B10 x SGR | 2 | 269U20b | 4105L19b10 |
| 2, CO only | B10 x SGR | 2 | 402U19b | 3044L18b10 |
| 1, NCO only * | B10 x SGR | 2 | 269U20b | 4105L18sgr |
| 2, NCO left | B10 x SGR | 2 | 269U20b | 2107L17b10 |
| 2, NCO right | B10 x SGR | 2 | 2092U16b10 | 3044L18sgr |
| 1 | B10 x R209 | 1 | 269U20 | 4623L20 + 4623L20b |
| 2, CO | B10 x R209 | 1 | 402U19 | 3402L20b |
| 2, NCO | B10 x R209 | 1 | 2092U16R209 | 3402L20 |
| 2, left | B10 x R209 | 1 | 269U20 | 2107L16R209 |
| 1 | B10 x R209 | 2 | 269U20b | 4623L20 + 4623L20b |
| 2, CO | B10 x R209 | 2 | 402U19b | 3402L20 |
| 2, NCO | B10 x R209 | 2 | 2092U16b10 | 3402L20b |
| 2, left | B10 x R209 | 2 | 269U20b | 2107L16b10 |
| 1 | B10.A x SGR | 1 | 6452U22A | 4623L20 + 4623L20b |
| 2, CO | B10.A x SGR | 1 | 6532U20A | 3402L20 |
| 2, NCO | B10.A x SGR | 1 | 2092U16R209 | 3402L20b |
| 2, left | B10.A x SGR | 1 | 6532U20A | 2107L16R209 |
| 1 | B10.A x SGR | 2 | 6452U22S | 4623L20 + 4623L20b |
| 2, CO | B10.A x SGR | 2 | 6534U18S | 3402L20b |
| 2, NCO | B10.A x SGR | 2 | 2092U16b10 | 3402L20 |
| 2, left | B10.A x SGR | 2 | 6534U18S | 2107L16b10 |
| 1 | R209 x SGR | 1 | 2160U18b10 | 4623L20 |
| 2 | R209 x SGR | 2 | 2176U17b10 | 3044L15sgr |
| 1 | R209 x SGR | 2 | 2161U17sgr | 4623L20b |
| 2 | R209 x SGR | 2 | 2176U17sgr | 3044L14b10 |

* In B10 x SGR, the allele-specificity of some primers was not sufficient to detect simultaneously CO and NCO in the orientation 2 (SGR-B10 CO and NCO on chromosome SGR). Therefore, CO and NCO have been detected independently in dedicated experiments.
